# Modelling and Analysis of the Effect of EDM-Drilling Parameters on the Machining Performance of Inconel 718 Using the RSM and ANNs Methods

**DOI:** 10.3390/ma15031152

**Published:** 2022-02-02

**Authors:** Magdalena Machno, Andrzej Matras, Maciej Szkoda

**Affiliations:** 1 Department of Rail Vehicles and Transport, Faculty of Mechanical Engineering, Cracow University of Technology, 31-155 Cracow, Poland; maciej.szkoda@pk.edu.pl; 2 Department of Production Engineering, Faculty of Mechanical Engineering, Cracow University of Technology, 31-155 Cracow, Poland; amatras@pk.edu.pl

**Keywords:** difficult-to-cut material, Inconel 718, electrical discharge machining, response surface methodology, artificial neural networks

## Abstract

Electrical Discharge Machining (EDM) is one of the most efficient processes to produce high-ratio micro holes in difficult-to-cut materials in the Inconel 718 superalloy. It is important to apply a statistical technique that guarantees a high fit between the predicted values and those measured during analysis of test results. It was especially important to check which method gives a better fit of the calculated result values in case they were relatively small and/or close to each other. This study developed models with the use of the response surface methodology (RSM) and artificial neural networks (ANNs). The aim of the study was comparison between two models (RSM and ANNs) and to check which model gives a better data fit for relatively similar values in individual tests. In all cases, the neural network models provided a better value fit. This is due to the fact that neural networks use better fitted functions than in the case of the RSM method using quadratic fitting. This comparison included the aspect ratio hole and the thickness side gap data, the values of which for individual tests were very similar. The paper reports an analysis of the impact of parameter variables on the analyzed factors. Higher values of current amplitude, pulse time length, and rotational speed of the working electrode resulted in higher drilling speed (above 15 µm/s, lower linear tool wear (below 15%), higher aspect ratio hole (above 26), lower hole conicity (below 0.005), and lower side gap thickness at the hole inlet (below 100 µm).

## 1. Introduction

In the manufacture of components for aero-gas turbine engines, efficient techniques for producing long micro holes are particularly desirable [1]. There are about 40,000 such holes (with diameters in the range of 0.3–5 mm and a ratio of the hole length to its diameter of up to 600:1 [2]) that are made in the turbine blade structure [3]. In the case of the aerospace industry, high-ratio micro holes (more than 100) must be characterized by high quality and dimensional and shape accuracy [4,5]. There are still no effective methods for making such micro holes in modern alloys and superalloys. Currently, one of the most effective techniques for drilling micro holes in these materials is electrical discharge drilling/machining (EDD/EDM) [6,7].

Particularly in the case of machining the Inconel 718 superalloy, most researchers prefer the electrical discharge drilling process [8]. This is due mainly to the mechanical properties of the Inconel 718 superalloy (high toughness, high hardness, poor thermal properties) which make it significantly more difficult to process using conventional methods [9]. However, due to the thermophysical properties of this superalloy (thermal conductivity—11.4 (W/(m×°K)), electrical resistivity—121 (µΩ×cm), specific heat capacity—435 (J/(kg×°K)), melting point—1483.15–1617.15 (°K), density—8.19 (g/cm^3^) [10,11]) it is hard to make high-ratio micro holes in this material, including in the case of electrical discharge drilling [12,13]. A significant limitation is the accumulation of eroded particles at the bottom of the hole as a result of difficulties in flushing the interelectrode gap area [14]. Accumulation of erosion products at the bottom of the hole contributes to arc discharges and short circuits, as well as secondary/abnormal discharges between the debris and the hole wall and between the debris and the electrode side surface [15]. These discharges are an undesirable phenomenon that reduces the accuracy of the hole geometry and causes increased wear of the working electrode [16].

The phenomena present in the machining area and impact of the Inconel 718 properties on the EDD process means that results analysis can be difficult. Research is still being conducted to improve performance characteristics of the EDD process, i.e., to increase the material removal rate (MRR) and simultaneously decrease the tool wear rate (TWR) or improve the surface roughness (*SR*) [17,18,19]. One of the ways to learn more about the influence of process parameters and to further understand the phenomena present in the interelectrode gap area is to analyze the relationship between the input and output factors, obtained by applying mathematical and statistical techniques.

Currently developed models describing the relationships between the input and resulting factors are also used to optimize the machining process parameters [20,21]. The most commonly used mathematical and statistical techniques include multiple regression and design of experiments (DOE) [22], the analysis of variance (ANOVA) [23], the response surface methodology (RSM) [24], Taguchi method [25], grey relational analysis (GRA) [12], artificial neural networks (ANNs) [26,27], neuro-fuzzy approach [22], and also their combinations [28,29]. For analyzing the effects of electrical discharge machining process parameters on performance factors, as well as developing empirical models, the RSM has been the most widely used technique in recent years [30]. It includes a set of mathematical and experimental techniques that require sufficient experimental data to analyze the problem and develop mathematical models that consider the dependencies of several input variables and the resulting factors [27,31,32]. This feature is important because the EDM process usually analyses the influence of several input factors (usually three, four or more), which can include: open circuit voltage, pulse-on-time, pulse-off-time, discharge current, duty cycle, working-fluid flushing pressure, electrode, electrode polarity, electrode material, and initial interelectrode gap [20].

In order to check the fit of the resulting models using statistical techniques, some researchers analyze the results using several methods at the same time. In [28], the authors suggest that using the RSM technique to analyze the effects of process parameters (peak current, electrode rotation speed, and peak voltage) on the MRR and the diametral overcut (DOC), made it possible to obtain an effective model in a short time. This approach is more appropriate due to the many factors affecting the process and still unknown phenomena present in the machining gap area. However, [29] presents a predictive model for wire electrical discharge machining of the Inconel 718 superalloy with the use of the RSM and ANN techniques. The analysis of the results showed that the model obtained by applying an artificial neural network provided more accurate and reliable predicted values of the analyzed resulting factors, compared to the RSM method. In addition, the lower value of the mean square error for the ANNs (1.49%) compared with the RSM (5.71%) confirms a better fit of the neural network model. In [27], on the other hand, an analysis of the relationship between the influence of electrical discharge drilling process parameters (discharge current, dielectric liquid pressure, and electrode rotational speed) on the resulting factors (machining rate, electrode tool wear, average over-cut, and taper angle) was carried out using methods such as the RSM, ANNs and the regression analysis (RA). But, the analyzed process parameters do not include all parameters most affecting the machining performance, such as pulse-on-time and pulse-off-time. For this reason, the developed models do not present sufficient information concerning optimization. The analysis of the models which were obtained demonstrated that the models generated with the use of the ANNs and RA can be successfully used to effectively predict results in the case of micro-drilling in AISI 304 stainless steel. In this case, to develop the models with the use of ANNs, the Levenberg–Marquardt algorithm was applied. The ANNs were trained with the use of 21 test outputs, and then it was checked and tested with the use of six test outputs. Moreover, in [22], the ANN technique which was applied provided a model describing the dependence of input factors on the resulting factors in the wire-EDM process of Inconel 718. To determine their models, the neural network was trained based on 81 experimental tests and each of its corresponding classes. In this case, the neural network structure involved 10 hidden layers. The ANN models were developed with an overall accuracy of 90%. However, the number of experimental tests is relatively large which extends the requirements for obtaining data to use in optimization. As above, it can be noted that neural networks can be defined in various ways and can be trained with the use of different amounts of data. The authors of [33] used two methods (RSM and ANNs) to determine the relationship between the input and resulting factors of electrical discharge drilling of micro holes in the Inconel 718 superalloy. However, the analyzed input parameters did not include the main process parameters affecting the response factors. In the analysis, there was a lack of influence parameters such as time-on-time, pulse-off-time and current amplitude. The analysis of the results showed a sufficient fit between the values calculated on the basis of both methods’ models compared to the measured values. Furthermore, the authors noted that neural networks can calculate the predicted values from experimental values with a high fit (for ANN models with 4-9-3 architecture). It is worthwhile to underline that the developed models have an application in this concrete case.

Accordingly, it is challenging to develop an adequate model to describe all the relationships and phenomena associated with making micro holes with the use of the electrical discharge drilling process. Therefore, any additional research and models developed using various statistical and mathematical techniques provides an important contribution to improving the process performance. In addition, the complexity of phenomena accompanying the removing allowance in the EDD process influences the development of a good model. From the reason, it is important to check several statistical methods when developing models based on the experimental data.

When modeling processes that use complex physical phenomena, the use of RSM, which enables the creation of models based on a polynomial quadratic function, is insufficient in many situations. The applied models using ANNs are built on the basis of time-consuming experimental research involving the performance of a large number of tests and measurements. The present study proposes the use of ANN modeling with a simple architecture consisting of several neurons in one hidden layer, combined with the use of a research plan. This approach is characterized by the possibility of using the advantages of modeling with the use of ANNs and a reduced amount of experimental studies accompanying modeling as compared with the use of the RSM. The analysis was developed on the basis of the data obtained from experimental studies of the electrical discharge drilling process in the Inconel 718 superalloy, including the influence of process parameters on the resulting factors, directly affecting the material removal rate (open voltage, pulse time, current amplitude) and the parameters related to the flow of the fluid through the interelectrode gap area (inlet dielectric pressure and tube-electrode rotation), on performance factors (drilling speed and linear tool wear), and geometric features of micro holes (aspect ratio hole, hole conicity, side gap thickness). The resulting data involved the effect of five input variables on the five resulting factors. In addition, values of the aspect ratio holes, the hole conicity and the side gap thickness are relative small and/or slightly different from each other. It is important to determine which method is preferable in order to obtain better fitted models for these output factors.

## 2. Materials and Methods

### 2.1. Selection of the Workpiece and Tool Material

The experimental study used the Inconel 718 superalloy as the workpiece material. It is amongst the materials most commonly used for turbine blades in jet engines, which was the determining factor for its choice. The dimensions of the specimen in which micro holes were made by electrical discharge drilling were 30 mm × 25 mm × 10 mm. The holes were drilled with a tube-shaped copper electrode with an outer diameter of 400 µm and inner channel diameter of 215 µm. According to [34], this type of working electrode is the best tool for electrical discharge drilling of micro holes. Table 1 shows the chemical composition of the Inconel 718 superalloy.

### 2.2. Experimental Procedure

The experimental studies were used to determine the effect of selected process parameters on the resulting factors (Table 2). In the electrical discharge drilling process, performance features such as drilling speed and linear tool wear have the greatest influence on the technological performance of the process. However, in the analysis of the geometry of high-ratio micro holes, the resulting factors such as hole aspect ratio, hole conicity, the thickness side gap are relevant.

**Figure 1:**
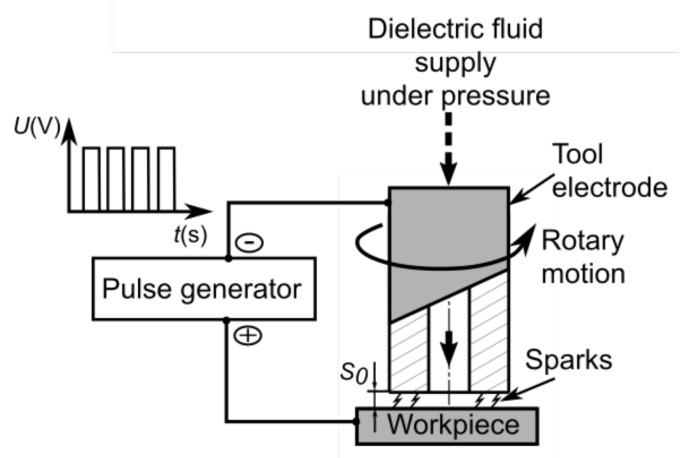
Scheme of the electrical discharge drilling process [13].

During the process, the electrodes (workpiece and working electrode) were connected to an electrical pulse generator. Between the electrodes there is a narrow interelectrode gap with an initial value. In addition, the tool electrode was performing rotary movement. The working fluid was flushed down under pressure to the gap area through the electrode channel (Figure 1). Table 3 presents a description of the test conditions and the applied constant values of the non-analyzed process parameters.

The values of the analyzed resulting factors were defined as follows:

- The drilling speed (*DS*) was calculated from the following equation [35]:

*DS = hole depth/drilling time,* (1)

where, for the through hole, its depth was equal to the working specimen height, i.e., 10 mm, while for the blind hole, the hole depth was defined as the value of the working electrode immersion from the initial machined surface.

- The linear tool wear (*LTW*) was calculated according to the formula

*LTW = (shortening of the electrode/hole depth)* × 100% (2)

To measure the shortening of the working electrode, the contact point of the electrode with the workpiece was measured before each test. The coordinates of the contact point were read; then, after processing, the working electrode was re-positioned at the contact point and the coordinates were read. The difference between the *Z*-axis coordinate value from the contact point before and after machining is the shortening value of the working electrode.

- The aspect ratio hole (*AR*) was given by the following equation [36]:

*AR* = *hole depth*/*average of the hole diameter* (3)

Each diameter (entrance and exit) was measured ten times, and the value of a given diameter was the average of these ten measurements. The average of the hole diameter constituted the average value from the average entrance diameter and the average exit diameter. In the case of a blind hole, the outer diameter of the tool electrode was used as the average exit diameter. For these measurements, a K-401 stereo microscope was used (Motic, Richmond, BC, Canada).

The hole conicity (HC) was calculated as difference between the average entrance and exit diameter divided by two and divided by the hole depth [35]

*HC* = (*entrance diameter* − *exit diameter*)/(2 × *hole depth*) (4)

- The thickness of the side gap (*SG*) was calculated according to the below formula [36]

*SG* = (*entrance diameter* − *diameter of the tool electrode*)/2 (5)

**Figure 2:**
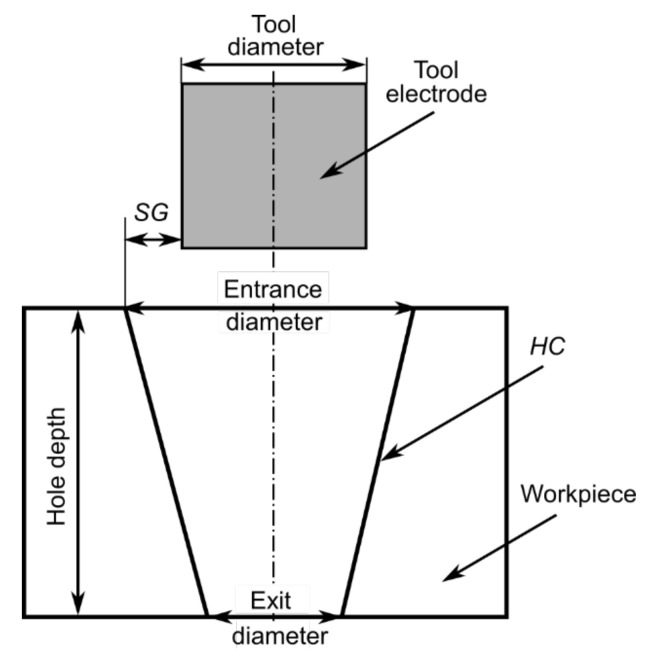
Measurement of the hole geometry.

The estimated hole geometry for *SG* and *HC* is additionally is shown in Figure 2.

Table 4 shows the used process parameters and their levels. The experimental research was carried on the prototype machine tool. The changeable values for the open voltage were in the range 60–120 V with the change possibility by 20 V. For this reason four variable values were used, such as 60, 80, 100, and 120 V. For the “0” level, the estimated values of the open voltage were rounded and closed to the higher value; therefore, more tests with *U* = 100 V were used in the adopted test plan.

In the investigation, the values of input factors were selected to allow stable and repeatable electrical discharge drilling tests. The experimental investigation was performed based on the adopted study plan. The measurements of the figures that were obtained (input diameter, output diameter, the shortening of the electrode) were repeated five times, and then the most extreme values were rejected, and the remaining ones were analysed. These assumptions provided more information about the impact of the changes in the input values on the resulting value. Table 5 presents the experimental research plan and the average values of the resulting factors, calculated on the basis of Formulas (1)–(5). The experimental tests were carried out according to the theory of the experiment using a rotatable study plan that included 31 experiments. The experimental tests consisted of 26 various tests and five repetitions in the center of the investigation plan (in Table 5 marked as *C*—the 27 test). As a result of the experiment, 31 micro holes were made. Thus, 93 data points were used to create the model for one analyzed resulting factor.

Table 6 presents the values of standard deviation for the measured resulting data.

### 2.3. Mathematical Modelling of the Research Object

Using the Response Surface Methodology (RSM) and Artificial Neural Networks (ANNs), models were created to describe and to determine the relationship between the process parameters and the resulting factors. Models for both methods were created using the same data (Table 5). The models were created, and the statistical analysis of the results was carried out using the STATISTICA programme (13.1, StatSoft, Tulsa, OK, USA).

Accordingly to Dong et al. [29] and Gangil et al. [20] the RSM method is one of the effective and the most frequently used methods for analyzing the influence of process parameters on the output factors. This information influenced the choice of the RSM method for use in this paper. On the other hand, the choice of the ANN method was based on the enormous possibilities of this method, which is confirmed by Sarikava et al. [27] and Lalwani et al. [29].

#### 2.3.1. Development of the RSM Models

In the RSM technique, a relationship is formed between the desired response and the independent input variables (*U*, *t_on_*, *I*, *p*, *n*), which can be represented as follows:

*Y* = *f*(*U*, *t_on_*, *I*, *p*, *n*) ± ε (6)

where *Y*—the desired response, *f*—the response function (or response surface) and *ε*—the fitting error, also called the residual error, are the experimental error measures. In the analysis procedure, an approximation of *Y* was suggested by use of the fitted second-order polynomial mathematical model, also determined as the quadratic model. This model (the research object function) might be expressed as follows:

(7) $$Y=\beta_{0}+\sum_{i=1}^{k}\beta_{i}x_{i}+\sum_{i=1}^{k}\beta_{ii}x_{i}^{2}+\sum_{i<j}^{k}\beta_{ij}x_{i}x_{j}\pm \varepsilon \;$$

where *k* is the amount of input process parameters (in the analysed case it equals 5); *x_i_* refers the input variables to the EDM parameters under study, *x_i_*^2^ and *x_i_x_j_* are the squares and interaction terms, respectively. *β_0_* is a constant coefficient and *β_i_*, *β_ii_*, *β_ij_* represent the coefficients determined by linear, quadratic, and cross product phrases, respectively [37]. *Y* was the analysed output variable.

**Figure 3:**
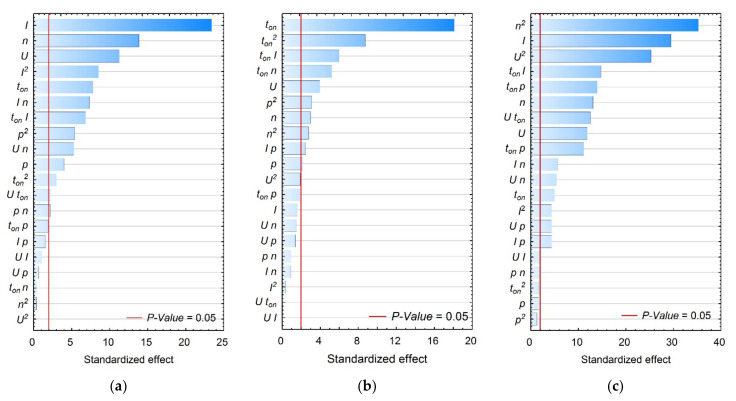

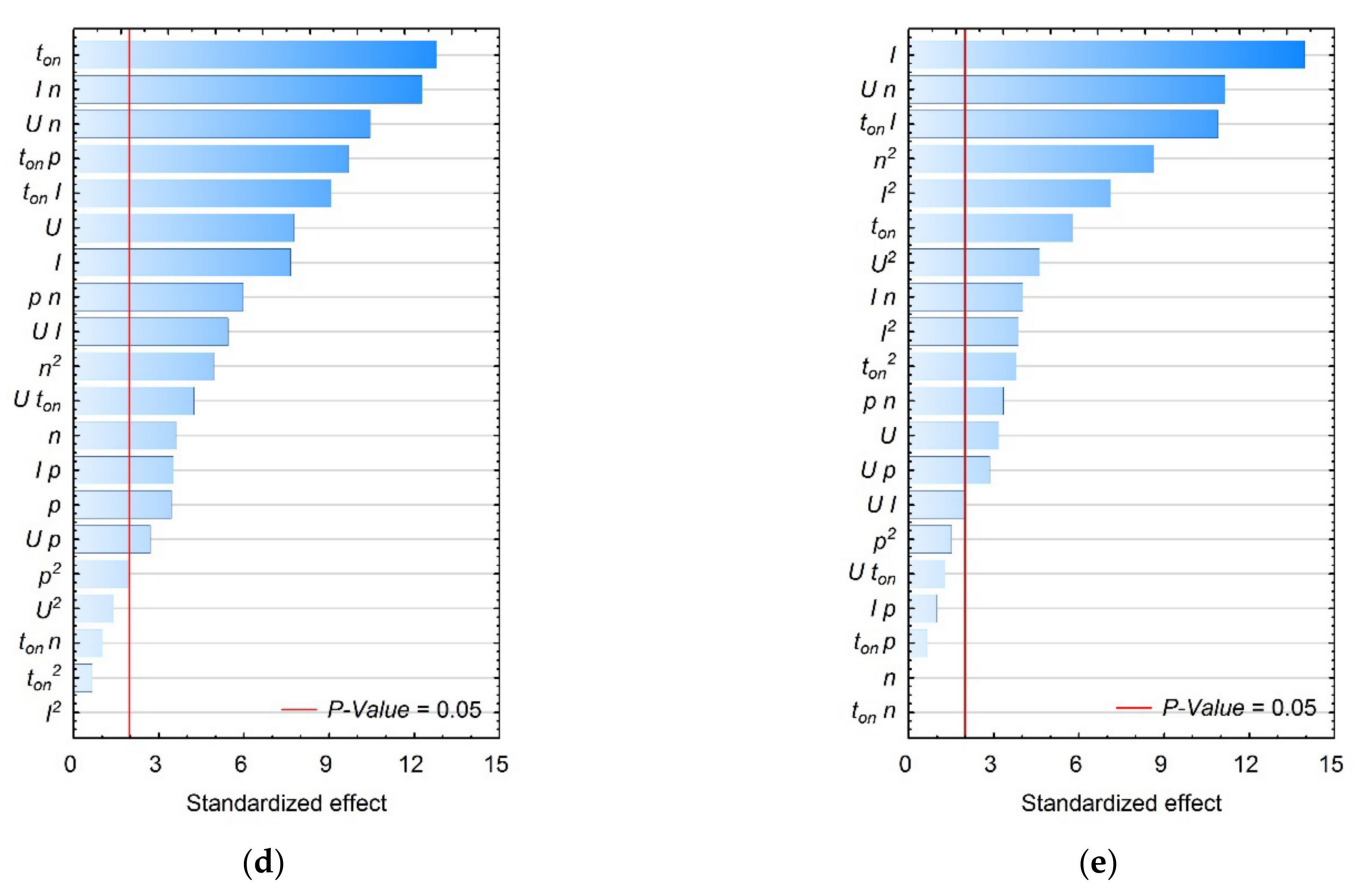
Pareto charts of the characterized effects (response is strength “alfa” = 0.05) for: (**a**) *DS*, (**b**) *LTW,* (**c**) *AR*, (**d**) *HC*, (**e**) *SG*.

The Analysis of Variance (ANOVA) was employed to statistically reduce the importance of the factors. For the RSM models which were obtained, significant variables were assumed for “*p-value*” < 0.05 (i.e., *α* = 0.05, or 95% confidence). After eliminating non-significant factors, the functions of the study object for resulting data took the form shown in Equations (8)–(12), respectively. Below, Figure 3a–e includes the Pareto diagrams, which characterize the statistical significance of the analysed input parameters on the obtained test results. Tables presenting the ANOVA results for *DS*, *LTW*, *AR*, *HC*, and *SG*, respectively, are included in the Supplementary Materials.

*DS* (µm/s) = 36.67 − 0.017 *U* + 0.0003 *t_on_* − 11.89 *I* − 0.45 *p* −0.047 *n* − 0.00001 *t_on_*^2^ + 1.41 *I*^2^ + 0.004 *p*^2^ − 0.0001 *U t_on_* + 0.0004 *U n* + 0.004 *t_on_ I* + 0.01 *I n* − 0.0002 *p n*, (8)

*LTW* (*%*) = 77.67 − 0.07 *U* + 0.097 *t_on_* + 14.14 *I* + 1.747 *p* + 0.004 *n* − 0.0001 *t_on_*^2^ − 0.01 *p*^2^ − 0.0001 *n*^2^ − 0.012 *t_on_ I* + 0.0001 *t_on_ n* − 0.11 *I p*, (9)

*AR* = − 22.82 + 1.0007 *U* − 0.019 *t_on_* − 2.07 *I* − 0.21 *p* + 0.056 *n* − 0.0058 *U*^2^ + 0.34 *I*^2^ − 0.0002 *n*^2^ − 0.0002 *U t_on_* + 0.002 *U p* + 0.0002 *U n* + 0.004 *t_on_ I* + 0.0003 *t_on_ p* + 0.00001 *t_on_ n* − 0.029 *I p* + 0.004 *I n*, (10)

*HC* = − 0.026 − 0.0007 *U* + 0.00008 *t_on_* − 0.0072 *I* + 0.0002 *p* + 0.0004 *n* + 0.0000001 *n*^2^ + 0.0000001 *U t_on_* + 0.0002 *U I* + 0.000005 *U p* − 0.000002 *U n* − 0.00002 *t_on_ I* − 0.000001 *t_on_* ∙ *p* + 0.00014 *I p* − 0.00005 *I n* − 0.000002 *p n*, (11)

*SG* (µm) = − 439.58 − 0.75 *U* + 0.49 *t_on_* + 209.08 *I* + 2.562 *p* + 0.50 *n* + 0.04 *U*^2^ − 0.00001 *t_on_*^2^ − 20.65 *I*^2^ + 0.001 *n*^2^ − 0.04 *U p* − 0.015 *U n* − 0.13 *t_on_ I* − 0.101 *I n* + 0.006 *p n*. (12)

#### 2.3.2. Development of the ANN Models

Artificial Neural Networks provided another method for determining the influence of input factors on the resulting factors. During the analyses with the application of neural networks, a multilayer perceptron (MLP) network was used. Networks of this type are successfully used to analyze complex phenomena of a nonlinear nature which are described by data characterized by a high noise level. The use of neural networks also enables the analysis of processes described with a limited amount of data [38]. Using a more complex method (e.g., neural networks) may lead to better results. A MLP neural network undergoes supervised learning based on a historical set of observations. Learning instances are presented at the network input. The result obtained at the output is compared with the pre-set value.

**Figure 4:**
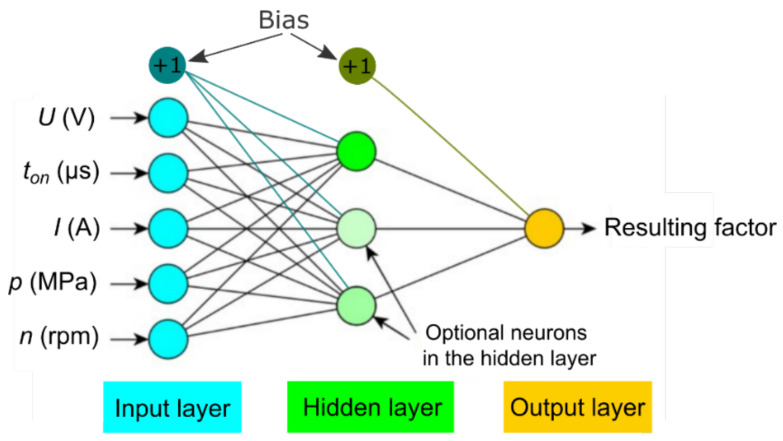
Neural network model.

In the case under analysis, the network learning process consisted of an iterative adjustment of the weights to minimize the pre-set error function comparing the network output with the pre-set value. Thus, the weights acted in the model as parameters optimized in the learning process. A number of neural networks were tested during the analyses. All networks consisted of three layers (Figure 4). All topologies of the tested networks included five neurons in the input layer and five input variables (predictors) were introduced. The networks were used to analyze one output variable at a time, so in the output layer there was one neuron. The architecture of the tested networks consisted of a single hidden layer. The number of neurons in the hidden layer and the types of activation functions in the hidden and output layers were selected experimentally. This permits generation of networks with a simple architecture and additionally prevents the phenomenon of over-fitting of the networks to the analyzed data.

In order to avoid the phenomenon of overfitting of the network to the analyzed data, the rule was used that the maximum number of neurons in the hidden layer was equal to half of the sum of the number of neurons in the input and output layers [38,39]. Therefore, networks with one to three neurons in the hidden layer were tested. Additionally, the phenomenon of overfitting of the network to the analyzed data was eliminated by analyzing the created models. 

The neurons in the hidden and output layers were activated using linear, logistic, sigmoid, exponential, or hyperbolic tangent functions. The argument in the following functions was the aggregated signal combined with weights.

In the above-described manner, for each of the analyzed output parameters, a number of networks consisting of different numbers of neurons in the hidden layer and combinations of different activation functions in the hidden and output layers were generated.

During the process of the individual neural networks learning, 66 from 93 data points (22 holes) were used as learning data and the remaining 27 data points (9 holes) were used to test and validate data. The data was allocated so that the measurements for the same cases in the study plan were not used for both training and test or validation data. The data allocation into training, test and validation has been selected experimentally. The network learning used the Broyden–Fletcher–Goldfarb–Shanno optimization algorithm (BFGS). The assumed error function was the sum of squares (*E*)—equation.

(13) $$E=\sum_{i=1}^{N}{\left(y_{i}-yy_{i}\right)}^{2}$$

where: *y_i_*—analyzed value of the output variable, *yy_i_*—predicted value of the output variable, *N*—number of variables.

The process of selecting the optimal networks comprised several stages. The main evaluation criterion was the evaluation of the model performance error on the test set. In the second instance, the error on the test set was compared with the error on the learning set. Preference was given to networks with a low error on the test set whose performance was consistent on both sets. If more than one network had the desired properties, the network with a less complex architecture was selected. The basic parameters of neural networks selected for creating models are summarized in Table 7.

A sensitivity analysis of the created neural networks was performed to determine the significance of the input variables. The measure of neural network sensitivity is the quotient of the error obtained at the run of the network for the data set without one variable and the error obtained with use all variables. If the quotient of errors is more than 1, the variable is considered significant. The results of the sensitivity analysis of the neural networks are summarized in Table 8. Based on them, it can be concluded that all input variables are significant. A high value of the sensitivity factor means that the change in the value of the input parameter has a strong influence on the results obtained in the application of the neural networks, i.e., the calculation result. In Table 8, the highest values of the sensitivity coefficients for individual analyzed parameters are marked with an asterisk. In this way, the input parameter of the analyzed process was indicated, the change of which has the greatest impact on the calculated value of the output parameter. Table 9 presents the generated values of network weight for the resulting factors.

## 3. Results

Fitting the RSM and ANN Models to the Measured Values

The analysis of the influence of the electrical discharge drilling process parameters variables on the resulting factors using the RSM and ANN methods showed that the models created on the basis of neural networks in each case provided a better fit between the calculated and the measured values. This was especially the case for the aspect ratio hole, the hole conicity and the side gap thickness. This conclusion is also consistent with the analysis of the literature which was performed.

Table 10 shows the following values: Pearson correlation coefficients (*R*), average deviations (*AD*), relative square errors (*RSE*) and relative average deviations (*RAD*) obtained from the analysis of the models created using the RSM and ANN methods, for the *DS* and *LTW* parameters. For the ANN models, the analysis results are additionally presented by dividing the data into learning, testing and validation. The values of the Pearson correlation coefficients and the determined errors for the *AR* and *HC* are presented in Table 11, and the ones for *SG* in Table 12.

The calculated and measured values of the drilling speed parameter are characterized by a high fit (*R* = 0.97 for the RSM model and *R* = 0.97 for the ANNs model). Likewise, the calculated values for the aspect ratio hole and the thickness side gap using both models are characterized by a high fit to the measured data. The Pearson correlation coefficients were, for the *AR*: *R* = 0.96—RSM and 0.97 for ANNs, and for *SG*: *R* = 0.95—RSM and 0.98—ANNs, respectively. For the *LTW*, the calculated and measured values have a good fit (for the RSM model, *R* = 0.88 and for the ANN model *R* = 0.97). Similarly, the calculated and measured values for hole conicity are characterized by a good fit (*R* = 0.85—RSM and *R* = 0.96 for ANNs). In all cases, a better fit between the calculated and measured values was obtained for the models created using the ANNs.

**Figure 5:**
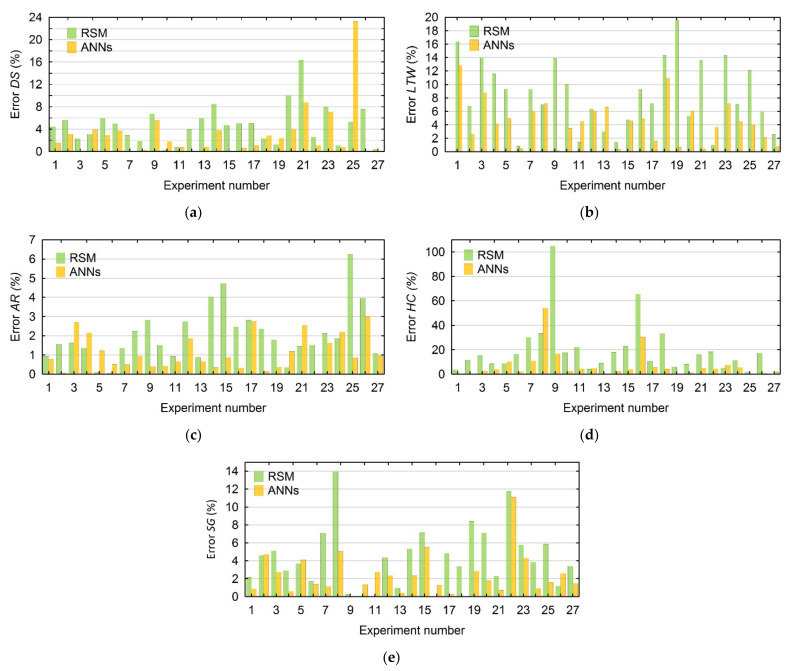
Comparison of the prediction error between the calculated values of: (**a**) *DS*, (**b**) *LTW,* (**c**) *AR*, (**d**) *HC*, (**e**) *SG*.

In addition, to compare the calculated values with the use of both methods, the prediction error is estimated according to Equation (14). The comparisons of error for values of *DS* and *LTW*, and *AR*, *HC*, *SG*, are shown in Figure 5a–e, respectively.

(14) $$\mathrm{Error}(\%)=\frac{Experimental\;values-Predicted\;values}{Experimental\;values}\;x100$$

**Figure 6:**
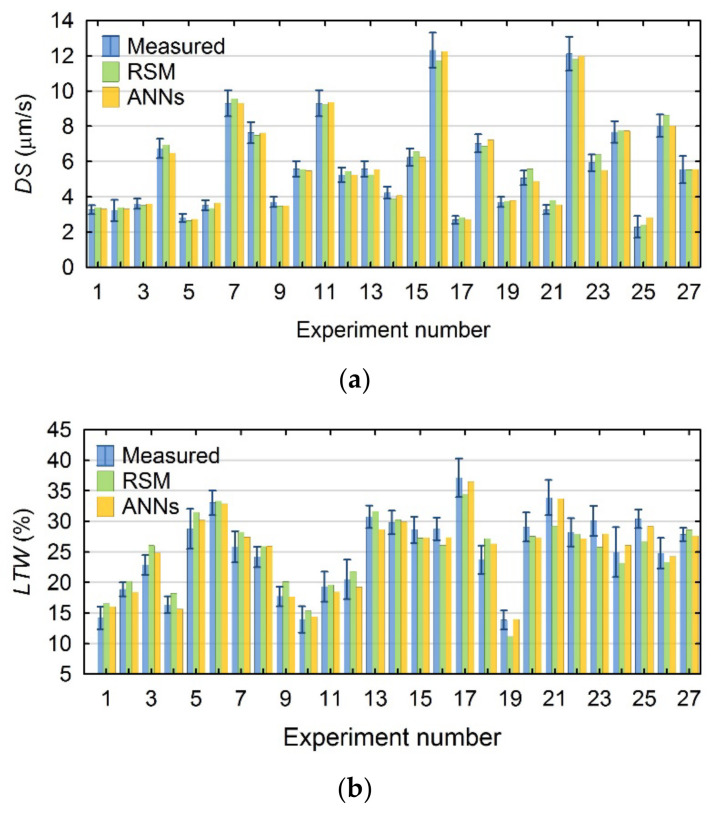
Summary of the values measured and calculated using the RSM and ANN models for: (**a**) *DS*, and (**b**) *LTW*.

For the analysis of the Pearson correlation coefficients and errors, the values calculated with the created models were compared with the average values measured for each of the test plan layouts. Figure 6a,b presents a comparison of these values for the *DS* and *LTW*.

The *DS* factor values calculated using both models, and the *LTW* parameter values calculated using the ANN model, are characterized by a high fit to the mean measured values. In some cases, the calculated values depart from the mean measured ones, but are within their standard deviations. In contrast, the *LTW* parameter values calculated with the RSM model for tests 3, 9, 19, 21, 23 and 25 differ significantly from the mean measured values. For 10 of the 27 tests in the plan, the values calculated using the RSM model are not within the range of standard deviations of the measured values.

When fitting the calculated values of the parameters characterizing the geometry of the hole using the RSM and ANN models, the following were obtained:

- for *AR*—a high fit with the use of both models;
- for *HC* and *SG*—a high fit with the use of the ANN models and a good fit with the use of the RSM models.

**Figure 7:**
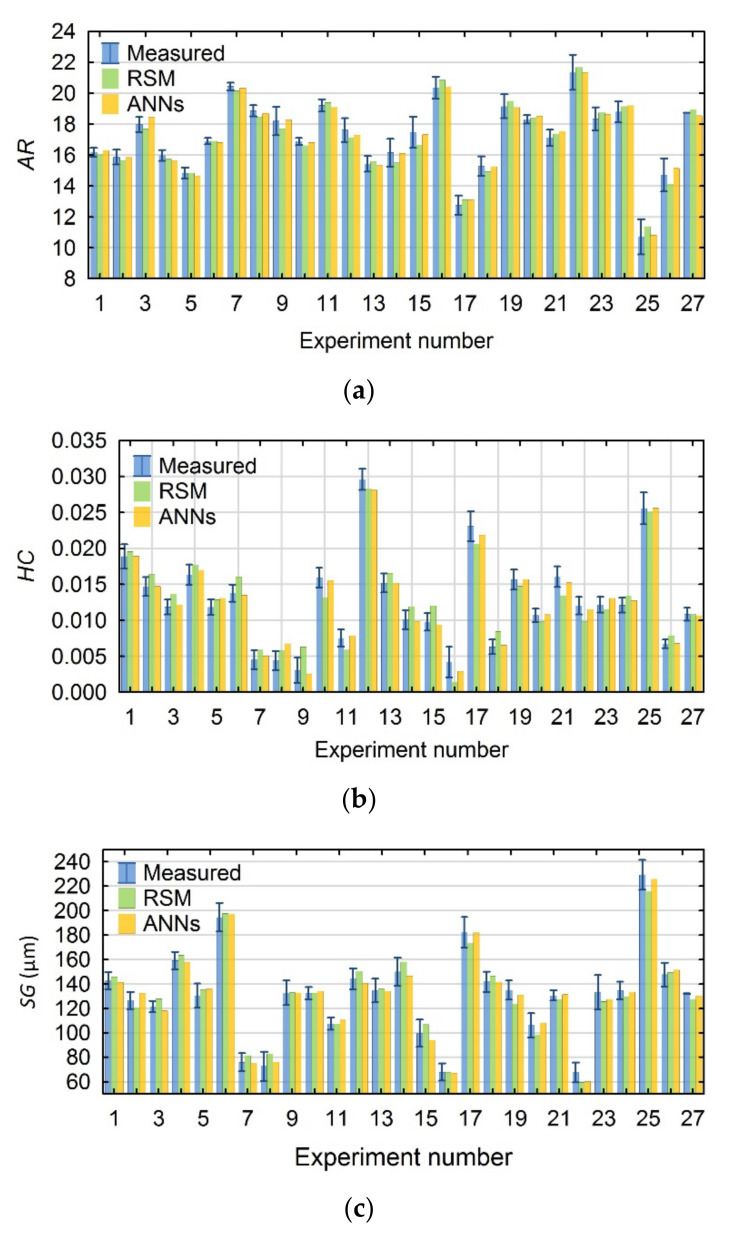
Summary of the values measured and calculated using the RSM and ANN models for (**a**) *AR*, (**b**) *HC*, and (**c**) *SG*.

For the *HC* parameter, for tests Nos. 9 and 6 of the research plan, the calculated values differ significantly from the mean measured ones. In addition, for tests Nos. 16 of the 27 plan layouts in the plan, the values of the *HC* parameter calculated with the RSM model are not within the standard deviation of the measured values. In addition, the *SG* values calculated with the RSM model depart from the mean measured ones, but are within their standard deviations. Figure 7a–c shows a comparison of the individual plan layouts for the *AR*, *HC* and *SG* resulting factors, including the measured values and those calculated using both methods.

## 4. Discussion

An Effect of the Process Parameters on the Resulting Data

This section compares the obtained relationships between the EDM process parameters and the result factors that were generated for the values calculated on the basis of experimental values, using the artificial neural network and response surface methodology methods. The included graphs showing relationships were selected from among over 100 generated graphs, which sufficiently present the differences between the obtained models.

**Figure 8:**
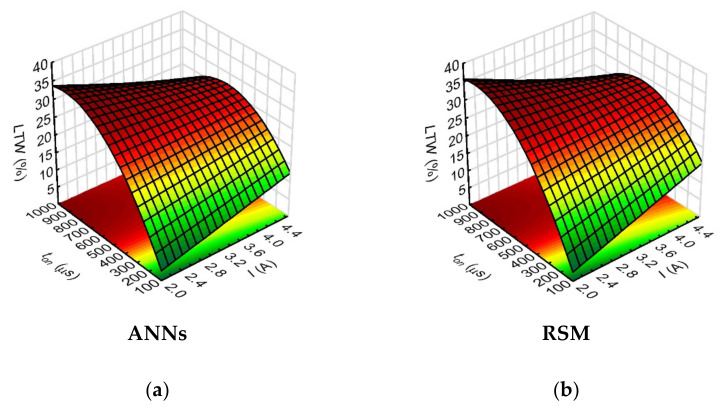
Impact of the input variables (*t_on_* and *I*) on the linear tool wear (*LTW*) with the use of: (**a**) ANNs, (**b**) RSM; *U* = 100 V, *p* = 70 MPa, *n* = 300 rpm.

The sensitivity analysis for the neural network model showed the greatest influence of just the pulse duration on the linear tool wear (the neural network sensitivity value for ton was 30.48). In this case, the fit of the models obtained using the RSM and ANN methods for the above-mentioned relationships was very similar (shown in Figure 8). The analysis of the results showed that the relationships which were obtained in the combinations of process parameters set with the pulse time length showed the highest linear electrode wear (*LTW* = 30–35%) for the highest pulse time values (*t_on_* = 700–999 µs).

The linear tool wear was most affected by the pulse time parameter, related to the influence of the thermal energy supplied to the test area, which is determined by this process parameter. However, in the case of machining the Inconel 718 superalloy, the energy distribution in the interelectrode gap region is disturbed due to the thermophysical properties of this material. Here, the low values of this superalloy properties such as thermal conductivity and thermal expansion coefficient have the greatest adverse effect. As a result, a significant amount of the heat present in the process was transferred to the working electrode material, which increased the electrode wear.

**Figure 9:**
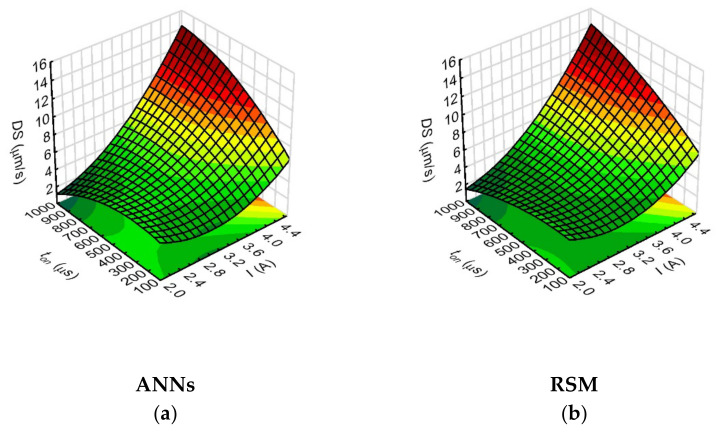
Impact of the input variables (*I* and *t_on_*) on the drilling speed (*DS*) with the use of: (**a**) ANNs, (**b**) RSM; *U* = 100 V, *p* = 70 MPa, *n* = 300 rpm.

**Figure 10:**
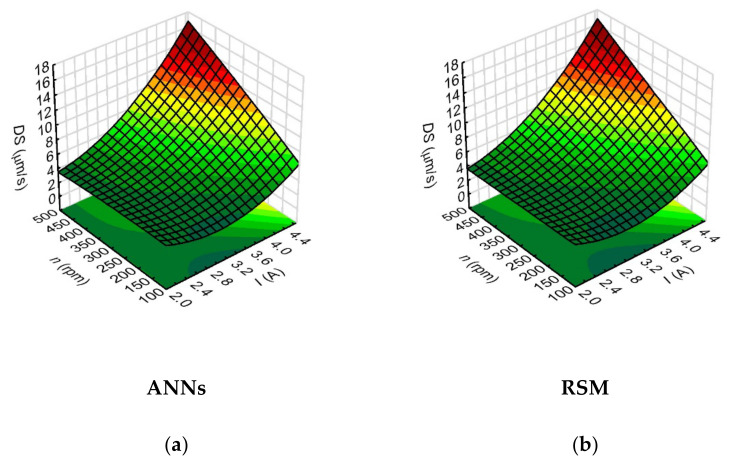
Impact of the input variables (*I* and *n*) on the drilling speed (*DS*) with the use of: (**a**) ANNs, (**b**) RSM; *U* = 100 V, *t_on_* = 550 µm, *p* = 70 MPa.

However, the analysis of the relationships showed that the drilling speed values were most strongly influenced by the analyzed process parameters such as the current amplitude (the network sensitivity index for *I* was 16.64). This is related to the energy of a single discharge, which is determined by the current intensity. Hence, the use of a higher value of current amplitude results in a higher single discharge energy, and the drilling speed then increases. The parameters that further influenced the drilling speed were the pulse time length (*t_on_*) (Figure 9) and the rotational speed of the working electrode (*n*) (Figure 10). During application of the maximum applied values of current amplitude (*I* = 4.65 A), the process parameters such as pulse duration (*t_on_* = 999 µs) and working electrode rotational speed (*n* = 500 rpm) provided higher value of drilling speed—above 15 µm/s. 

The pulse time length determines the amount of thermal energy supplied to the workpiece material. The heat energy causes partial evaporation and partial melting of the workpiece material and of the working electrode. The rotational speed of the working electrode, on the other hand, has a significant effect on the removal of erosion products from the interelectrode gap area, which provides stable drilling conditions. The *DS* (*I*, *n*) relationships (shown in Figure 10) confirm that the increase in the drilling speed occurred with an increase in the values of the process parameters: current amplitude and working electrode rotational speed.

Moreover, the analysis of the results indicated that the linear working electrode wear and the drilling speed were the least affected by the variable parameter of the initial fluid supply pressure. For the *LTW*, the network sensitivity index for this input variable was 1.21, and for the *DS* it was 3.28. In addition, for the drilling speed factor the calculated values by both methods provided similar predicted values. However, for linear tool wear, the RSM method, in some cases, yielded predicted values characterized by a relatively large error. Therefore, in this case, a better fit of the calculated values to the measured values was obtained by using the neural network model.

**Figure 11:**
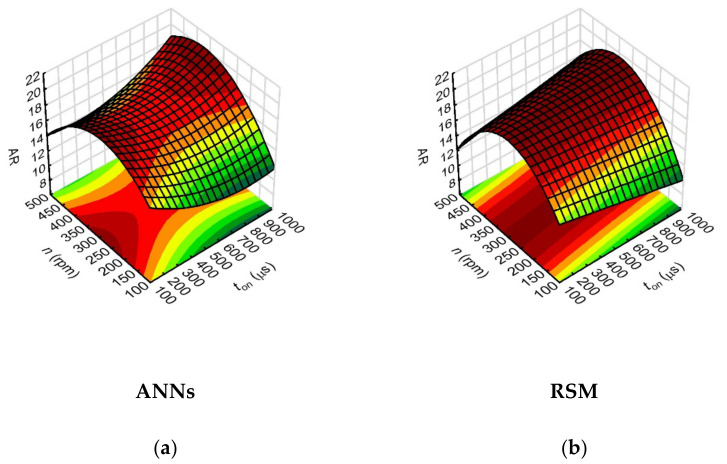
Impact of the input variables (*t_on_* and *n*) on the aspect ratio hole (*AR*) with the use of: (**a**) ANNs, (**b**) RSM; *U* = 100 V, *I* = 3.33 A, *p* = 7 MPa.

The analysis of the results showed that the aspect ratio hole (*AR*) values were mostly influenced by the working electrode rotational speed parameter (*n*) (ANNs—the highest value of the network sensitivity index for n, amounting to 13.84). For the n values used in the range of 200–400 rpm, the aspect ratio hole values were about 20 (Figure 11). In [40], it was also demonstrated that the rotation of the electrode is one of the best techniques to reach a high aspect ratio hole. Applied higher rotational electrode speeds also helps to rechange the working fluid for fresh at the machining gap area and to reduce the working fluid temperature during the interval pulse. The process can then run more stably, and efficient removal of eroded particles prevents unwanted electrical discharges.

Comparing the relationships obtained with the use of ANN and RSM methods, one can notice a different nature of AR changes for *t_on_* > 700 μs. If the calculated values are fitted by ANNs, the highest AR values of about 20 are obtained for *t_on_* = 900–1000 µs. On the other hand, the RSM method averaged the calculated data more. This shows the high sensitivity of neural networks in adjusting the calculated values to the experimental results. It is very important for the EDM process, because the change of the impulse time value range significantly influences the conditions and phenomena in the area of the inter-electrode gap.

**Figure 12:**
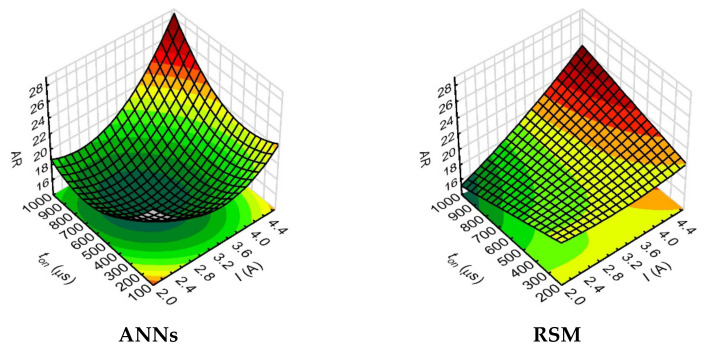
Impact of the input variables (*t_on_* and I) on the aspect ratio hole (*AR*) with the use of: (**a**) ANNs, (**b**) RSM; *U* = 100 V, *p* = 7 MPa, *n* = 300 rpm.

In the relationship analysis, the aspect ratio hole value was also observed to increase with the increase of the values of current amplitude and pulse time length. The analysis of the effect of process parameters, such as ton and *I*, on the aspect ratio hole and the drilling speed, showed very similar relationships. An increase in ton and *I* resulted in an increase in the *AR* and an increase in the *DS*. For the maximum applied values of these process parameters (*I* = 4.65 A and *t_on_* = 999 µs), the *AR* reached 26–28 (Figure 12), whilst the *DS* was 14–16 µm/s (Figure 9).

In the case of the *AR* rectorships, the fit of the models obtained using the ANN and RSM methods for the above-mentioned relationships were very different. The calculated values of ANN models indicate better fitting to the measured values of *AR*.

The analysis of the impact of the variables data on the hole conicity (*HC*) and the side gap thickness (*SG*), demonstrated the highest impact of the current amplitude and, further on, of the rotational speed of the working electrode. The sensitivity indices of the neural network for the influence of these process parameters were: 171.50 and 18.29 for *I*, and 107.38 and 13.68 for n, respectively, for *HC* and *SG*. Since the drilled micro holes were characterized by higher average values of the inlet diameter than the output diameter, the values of hole conicity were significantly influenced by the values of the inlet diameter determined by the side gap thickness at the hole inlet.

**Figure 13:**
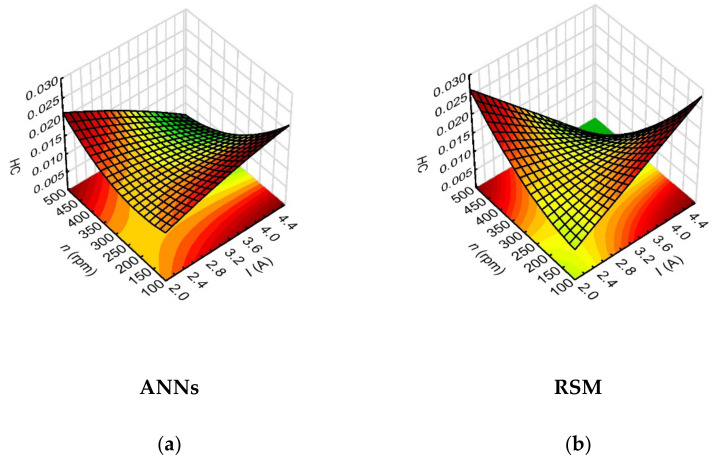
Impact of the input variables (*I* and *n*) on the hole conicity (*HC*) with the use of: (**a**) ANNs, (**b**) RSM; *U* = 100 V, *t_on_* = 550 µs, *p* = 7 MPa.

**Figure 14:**
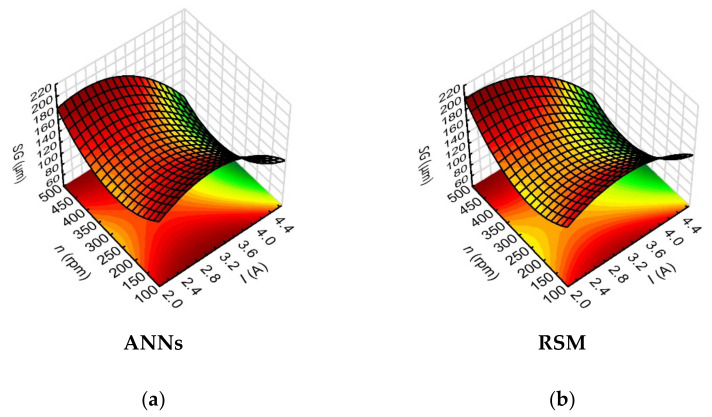
Impact of the input variables (*I* and *n*) on the side gap thickness (*SG*) with the use of: (**a**) ANNs, (**b**) RSM; *U* = 100 V, *t_on_* = 550 µs, *p* = 7 MPa.

**Figure 15:**
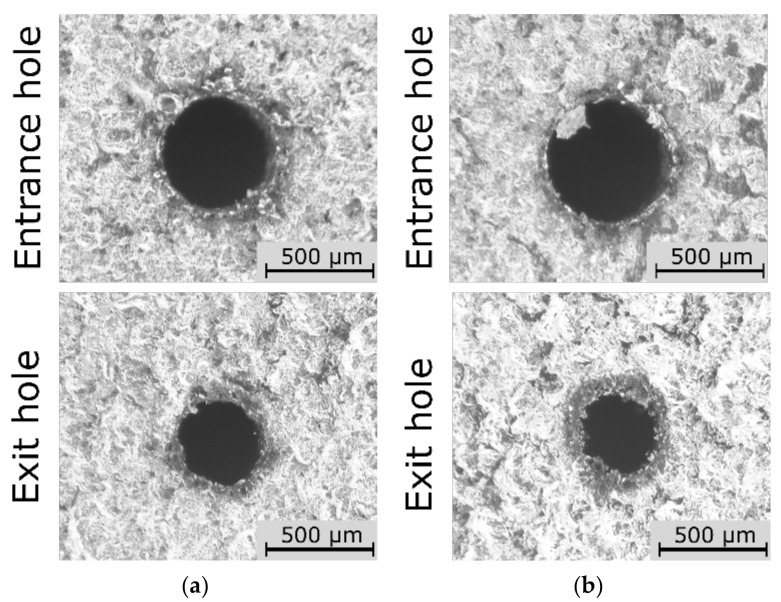
Images of the entrance and exit hole for the test: (**a**) *I* = 4.65 A and *U* = 100 V, *t_on_* = 550 µs, *p* = 7 MPa, *n* = 300 rpm; (**b**) *n* = 500 rpm and *I* = 3.33 A, *U* = 100 V, *t_on_* = 550 µs, *p* = 7 MPa.

The *HC* (*I*, *n*) relationship indicated that the lower *HC* values (below 0.006) were obtained for higher values of *I* = 4.00–4.65 A and higher values of *n* = 400–500 rpm (Figure 13). There was a similar relationship for higher values of *I* and higher values of *t_on_* = 800–999 µm—*HC* < 0.005. Similarly, the analysis of the results for the side gap thickness factor showed that the lower values of *SG* < 100 µm were obtained for *I* = 4.00–4.65 A and *n* = 400–500 rpm (Figure 14). This result is associated with sufficiently high thermal energy supplied to the workpiece material, and proper material removal. Higher values of *n*, on the other hand, ensured relatively efficient removal of the eroded particles. In order to confirm the higher dimensional and shape accuracy of the holes made with the use of the maximum value of the current amplitude and the maximum value of the working electrode rotational speed in the conducted tests, the relevant Figure 15a,b is shown below.

The *AR* calculated using the ANN and RSM methods did not always yield similar values. As a result, the relationships presented in the 3D plots differed in some cases. Here, a better fit of the model to the measured values was obtained using the ANN method. This is due to the fact that the model created with neural networks used a logistic function, which is not available in the RSM method. The neural networks use better fitted functions (tanh, exponential, logistical, linear) than in the case of the RSM method using quadratic fitting. In addition, neural networks use different functions in the hidden layer and the output layer (for example, the tanh function was used for the *DS* factor in the hidden layer, and the exponential function in the output layer, Table 7). On the other hand, the *SG* and *HC* values calculated from the two models provided similar predicted values. In addition, for factors related to the geometric characteristics of the micro holes, the initial liquid supply pressures used did not strongly affect the relationships which were obtained. This was also confirmed by the analysis of the neural network sensitivity indices for the *AR*, *SG* and *HC*. All the neural network sensitivity indices for these resulting factors reached the smallest values.

The analysis of the relationship between the process parameters and the result factors in this study showed that the ANN method provided a better fit of the calculated values of the result factors when their values for individual tests were relatively small or close to each other (as was the case with the data for aspect ratio hole and the thickness side gap). This is important for the EDM process used to produce microholes and the subsequent selection of the appropriate statistical method, particularly due to the fact that the analyzed result factors characterizing the geometry of the holes slightly differ from each other. In addition, the 3D figures (Figure 8, Figure 9, Figure 10, Figure 11, Figure 12, Figure 13 and Figure 14—ANNs) characterizing the models were created based on the 400 points calculation used in the models. On their basis, it can be concluded that there is no over-fitting phenomenon. However, determining the relationship between input and output factors provides important information when analyzing data. In this case, the selected statistical method should be appropriately sensitive and accurate, and the data analysis in this study showed that artificial neural networks are able to cope better than the response surface methodology method.

The analysis of the relationships which were obtained confirmed that the resulting factors of the electrical discharge drilling process are significantly affected by many process parameters. In addition, there are some disturbing factors, which also affect the process performance factors and geometric features of the micro holes. This shows that further experiments are still needed to better understand the phenomena present in the narrow interelectrode gap area, as well as in the side gap area near the bottom of the hole. The inability to monitor the processing area during electrical discharge drilling makes it important to analyze the influence of input factors on the resulting factors using statistical and mathematical methods.

## 5. Conclusions

This study uses techniques such as artificial neural networks and response surface methodology to analyze the influence of input variables on the resulting data, for data obtained from the Inconel 718 electrical discharge drilling tests. In the case of making micro holes by means of this process, an analysis of the impact of the input factors on the resulting factors is a difficult task to perform. As a result, developing a model that can guarantee, as far as possible, a high match between the calculated and the measured values is a challenge. This is especially difficult in case where the resulting values are relatively small and/or similar to each other. In this case, it was particularly difficult to determine the relationship when analyzing the data on output factors such as aspect ratio hole and the thickness side gap. For these result factors, the values for the individual trials in the plan did not differ significantly from one another. The comparison of the results of the methods used has shown that artificial neural networks in this case has a much better application than the response surface methodology method. The method of artificial neural networks allowed choice from a wide range of approximating functions, which enabled the possibility of creating a model characterized by a smaller error of fitting. This research also points to the learning potential of neural networks and the neural network models that guarantee a high match of the predicted values to the measured data.

However, the results analysis of effects of input variables on the resulting data, showed:

- the properties of Inconel 718 significantly affect the hole geometry (in some cases, the material can be removed inappropriately due to too low energy delivered to workpiece material);
- the parameters which mainly affected the resulting data were the current amplitude, the pulse time length, and the rotational speed of the working electrode;
- higher values of the above-mentioned parameters (4.00–4.65 A, 800–999 µs and 400–500 rpm, respectively), provided higher drilling speed (above 15 µm/s, lower linear tool wear (below 15%), higher aspect ratio hole (above 26), lower hole conicity (below 0.005) and lower side gap thickness at the hole inlet (below 100 µm).

Further experimental research is hence needed to determine neural networks models for wider ranges of values and additional variable parameters of the electrical discharge drilling process, and in particular for larger maximum values of the initial fluid supply pressure to the machining area.

## Figures and Tables

**Table 1:** Chemical composition of Inconel 718 (wt.%) [13].

Ni	Cr	Fe	Nb	Mo	Ti	Al	Co	Mn	C	Si	P
50.0–55.0	17.0–21.0	Balance	4.75–5.5	2.8–3.3	0.65–1.15	0.2–0.8	<1.0	<0.35	<0.08	<0.35	<0.015

**Table 2:** Process parameters and the analyzed factors.

Input Parameters	Resulting Factors
Open voltage, *U* (V)	Drilling speed, *DS* (μm/s)
Pulse time, *t_on_* (μs)	Linear tool wear *LTW* (%)
Current amplitude, *I* (A)	Aspect ratio hole, *AR*
Inlet dielectric fluid pressure, *p* (MPa)	Hole conicity, *HC*
Tube-electrode rotation, *n* (rpm)	Side gap thickness, *SG* (µm)

**Table 3:** Machining conditions and constant factors.

Description	Value/Characteristic
Initial interelectrode gap, *S_0_* (µm)	50
Drilling time of each hole, *t_drilling_* (min)	45 (or shorter in the case where the through hole was obtained faster)
Pulse off time, *t_off_* (µs)	550
Working fluid	Deionized water with an average electrical conductivity of 5 µS/cm

**Table 4:** Process parameters and their levels.

Parameter	Values Range	Levels				
*U* (V)	60–120	−2	−1	0/+1		+2
		60	80	100		120
*t_on_* (µs)	100–999	−2	−1	0	+1	+2
		100	325	550	775	999
*I* (A)	2.00–4.65	2.00	2.66	3.33	3.99	4.65
*p* (MPa)	5–9	5	6	7	8	9
*n* (rpm)	100–500	100	200	300	400	500

**Table 5:** Process variables and their resulting factors.

Exp. No.	Research Plan					Output Factors				
	*U* (V)	*t_on_* (µs)	*I* (A)	*p* (MPa)	*n* (rpm)	*DS* (µm/s)	*LTW* (%)	*AR*	*HC*	*SG* (µm)
1 *	80	325	2.66	6	400	3.25	14.20	16.18	0.0189	143
2 *	80	325	2.66	8	200	3.22	18.88	15.87	0.0147	126
3 *	80	325	3.99	6	200	3.59	22.84	17.98	0.0119	121
4	80	325	3.99	8	400	6.74	16.30	15.97	0.0163	159
5 *	80	775	2.66	6	200	2.81	28.76	14.84	0.0118	130
6 *	80	775	2.66	8	400	3.52	33.03	16.90	0.0138	194
7	80	775	3.99	6	400	9.30	25.84	20.44	0.0045	76
8	80	775	3.99	8	200	7.64	24.18	18.87	0.0044	73
9	100	325	2.66	6	200	3.70	17.68	18.21	0.0031	133
10	100	325	2.66	8	400	5.58	13.92	16.88	0.0159	132
11	100	325	3.99	6	400	9.30	19.29	19.22	0.0075	107
12	100	325	3.99	8	200	5.24	20.48	17.62	0.0296	144
13	100	775	2.66	6	400	5.58	30.73	15.43	0.0152	135
14	100	775	2.66	8	200	4.23	29.81	16.16	0.0101	150
15	100	775	3.99	6	200	6.25	28.60	17.48	0.0098	100
16	100	775	3.99	8	400	12.32	28.75	20.36	0.0042	68
17 *	60	550	3.33	7	300	2.69	37.11	12.76	0.0231	182
18	120	550	3.33	7	300	7.03	23.73	15.28	0.0063	142
19	100	100	3.33	7	300	3.70	13.85	19.16	0.0157	135
20	100	999	3.33	7	300	5.07	29.06	18.31	0.0107	106
21 *	100	550	2.00	7	300	3.27	33.85	17.12	0.0161	130
22	100	550	4.65	7	300	12.12	28.19	21.35	0.0120	67
23	100	550	3.33	5	300	5.93	30.07	18.35	0.0122	133
24	100	550	3.33	9	300	7.68	24.94	18.79	0.0121	135
25 *	100	550	3.33	7	100	2.29	30.38	10.72	0.0256	229
26	100	550	3.33	7	500	8.02	24.78	14.71	0.0067	148
27(*C*)	100	550	3.33	7	300	5.54	27.84	18.73	0.0109	132

* Indicates a blind hole.

**Table 6:** The values of standard deviation for the measured resulting factors.

Exp. No.	Standard Deviation				
	*DS*	*AR*	*HC*	*LTW*	*SG*
1	0.26	0.28	0.0017	0.92	7.07
2	0.61	0.47	0.0013	0.58	7.06
3	0.29	0.49	0.0010	0.81	4.70
4	0.54	0.36	0.0014	0.68	7.11
5	0.22	0.35	0.0011	1.64	10.02
6	0.28	0.20	0.0012	1.01	11.66
7	0.74	0.23	0.0013	1.28	7.49
8	0.61	0.36	0.0013	0.84	11.80
9	0.30	0.92	0.0018	0.80	10.12
10	0.45	0.22	0.0014	1.08	5.00
11	0.74	0.40	0.0012	1.24	5.00
12	0.42	0.77	0.0015	1.62	8.67
13	0.45	0.50	0.0013	0.91	9.57
14	0.34	0.89	0.0013	0.97	11.51
15	0.50	1.01	0.0012	1.08	11.13
16	0.99	0.70	0.0021	0.93	7.00
17	0.22	0.63	0.0021	1.58	12.48
18	0.51	0.63	0.0010	1.15	8.33
19	0.30	0.77	0.0014	0.77	7.87
20	0.41	0.27	0.0009	1.19	9.99
21	0.26	0.53	0.0014	1.44	4.29
22	0.97	1.14	0.0012	1.15	8.23
23	0.47	0.74	0.0011	1.23	14.14
24	0.61	0.68	0.0010	2.04	7.10
25	0.61	1.14	0.0022	0.77	12.18
26	0.64	1.08	0.0006	1.25	9.77
27	0.78	0.01	0.0009	0.54	0.21

**Table 7:** Selected parameters of chosen neural networks.

Resulting Factor	Number of Neurons in the Hidden Layer	Activation Function in the Hidden Layer	Activation Function in the Output Layer	Optimisation Algorithm
*DS*	3	tanh	exponential	*BFGS* 88
*LTW*	2	tanh	exponential	*BFGS* 29
*AR*	3	logistical	linear	*BFGS* 119
*HC*	3	tanh	linear	*BFGS* 142
*SG*	3	exponential	exponential	*BFGS* 72

**Table 8:** Neural networks’ sensitivity analysis for input variables.

Resulting Factor	Values of Sensitivity Analysis				
	*U*	*t_on_*	*I*	*p*	*n*
*DS*	6.44	7.19	16.64 *	3.28	7.23
*LTW*	3.10	30.48 *	2.32	1.21	1.54
*AR*	8.20	11.83	8.11	3.53	13.84 *
*HC*	17.69	15.30	171.50 *	3.65	107.38
*SG*	8.74	5.09	18.29 *	1.45	13.68

* Indicates the parameter mainly affecting the resulting factor.

**Table 9:** Network weights for *DS*, *LTW*, *AR*, *HC*, and *SG*.

Connection	Weight Values				
	*DS*	*LTW*	*AR*	*HC*	*SG*
*U* → hidden neuron 1	1.72	−0.20	−5.13	28.04	2.29
*t_on_* → hidden neuron 1	−1.71	−6.06	5.99	−17.48	3.42
*I* → hidden neuron 1	−5.02	−2.37	2.04	−50.84	5.54
*p* → hidden neuron 1	−2.25	0.89	3.54	−11.11	0.07
*n* → hidden neuron 1	−1.75	0.84	3.08	−18.67	−3.40
*U* → hidden neuron 2	0.25	−0.87	0.89	−1.35	−4.70
*t_on_* → hidden neuron 2	0.18	−3.59	−8.70	−2.39	1.84
*I* → hidden neuron 2	−1.93	−2.56	−6.96	7.40	1.70
*p* → hidden neuron 2	−1.16	0.62	−3.77	1.44	1.17
*n* → hidden neuron 2	−0.69	0.17	−0.45	−5.74	5.30
*U* → hidden neuron 3	4.58	-	−7.31	−0.82	0.85
*t_on_* → hidden neuron 3	4.26	-	−2.59	−2.39	3.82
*I* → hidden neuron 3	2.72	-	−3.47	10.07	7.72
*p* → hidden neuron 3	−0.55	-	−0.43	1.38	−0.13
*n* → hidden neuron 3	3.34	-	7.31	−9.11	−0.75
bias → hidden neuron 1	3.74	1.84	−4.63	39.93	−4.36
bias → hidden neuron 2	1.42	1.74	10.22	−0.25	−1.77
bias → hidden neuron 3	−7.20	-	7.14	−0.11	−6.18
hidden neuron 1 → response	2.07	−3.72	−1.09	0.36	0.23
hidden neuron 2 → response	−2.76	3.28	−1.19	3.39	0.03
hidden neuron 3 → response	1.18	-	1.22	−3.15	−0.33
bias → response	−1.75	−1.04	0.81	0.35	−0.92

**Table 10:** Analysis of the fit between the calculated and measured values for the *DS* and *LTW*.

Source	*DS*						*LTW*			
	RSM	ANNs				RSM	ANNs			
	All	All	Teaching	Test	Validation	All	All	Teaching	Test	Validation
*R*	0.97	0.97	0.97	0.98	0.97	0.88	0.97	0.97	0.96	0.97
*AD*	0.47	0.44	0.43	0.43	0.49	2.44	1.25	1.28	1.41	0.98
*RSE*	0.01	0.01	0.01	0.01	0.01	0.03	0.004	0.01	0.01	0.003
*RAD*	0.09	0.08	0.08	0.08	0.08	0.11	0.05	0.05	0.06	0.04

**Table 11:** Analysis of the fit between the calculated and measured values the *AR* and *HC*.

Source	*AR*						*HC*			
	RSM	ANNs				RSM	ANNs			
	All	All	Teaching	Test	Validation	All	All	Teaching	Test	Validation
*R*	0.96	0.97	0.97	0.97	0.96	0.85	0.96	0.97	0.96	0.96
*AD*	0.37	0.47	0.41	0.42	0.61	0.002	0.001	0.001	0.001	0.001
*RSE*	0.002	0.002	0.002	0.002	0.003	0.24	0.04	0.03	0.04	0.06
*RAD*	0.02	0.03	0.02	0.03	0.04	0.23	0.11	0.10	0.13	0.14

**Table 12:** Analysis of the fit between the calculated and measured data for *SG*.

Source		*SG*			
	RSM	ANNs			
	All	All	Teaching	Test	Validation
*R*	0.95	0.98	0.99	0.98	0.98
*AD*	10.14	5.80	5.88	6.83	4.36
*RSE*	0.01	0.004	0.004	0.004	0.002
*RAD*	0.08	0.05	0.05	0.06	0.04

## Data Availability

The data presented in this study are available on request from the corresponding authors. The data are not publicly available due to privacy.

## Supplementary Materials

The following supporting information can be downloaded at: https://www.mdpi.com/article/10.3390/ma15031152/s1. Table S1: ANOVA table for the *DS* and *LTW*; Table S2: ANOVA table for the *AR* and *HC*; Table S3: ANOVA table for the *SG*.
