# Supplementary material for: Modelling and Analysis of the Effect of EDM-Drilling Parameters on the Machining Performance of Inconel 718 Using the RSM and ANNs Methods
Source: Materials (Basel). 2022 Feb 2;15(3):1152. doi: 10.3390/ma15031152 (PMC8838809; doi:10.3390/ma15031152)
Supplement: Supplementary file 1 [file materials-15-01152-s001.zip › materials-1539634-supplementary(1).pdf]

Modelling and Analysis of the Effect of EDM-Drilling Parameters on the Machining Performance of Inconel 718 Using the RSM and ANNs Methods

Magdalena Machno [1,] *, Andrzej Matras [2] and Maciej Szkoda [1]

[1] Department of Rail Vehicles and Transport, Faculty of Mechanical Engineering, Cracow University of Technology, 31-155 Cracow, Poland; maciej.szkoda@pk.edu.pl

[2] Department of Production Engineering, Faculty of Mechanical Engineering, Cracow University of Technology, 31-155 Cracow, Poland; amatras@pk.edu.pl

* Correspondence: magdalena.machno@pk.edu.pl, Tel.: +48-12-374-36-56

The Analysis of Variance (ANOVA) was employed to statistically reduce the importance of the factors. For the RSM models which were obtained, significant variables were assumed for *P-Value* < 0.05 (i.e., α = 0.05, or 95% confidence). After eliminating non-significant factors, the functions of the study object for resulting data took the form shown in Equations (8)–(12) in the article, respectively. Tables S1–S3 present the ANOVA results for drilling speed (*DS*), linear tool wear (*LTW*), aspect ratio hole (*AR*), hole conicity (*HC*), and thickness of side gap (*SG*), respectively. Parameters *Seq SS* is a sum of squares, *DF* is a degree of freedom, *MS* is a mean square. The hypotheses of statistical significance of the influence of individual parameters were tested using the F and P statistics. The results of these tests were described using the *F-Value* and *P-Value* parameters.

Table S1. ANOVA table for the *DS* and *LTW*.

Source	*DS*					*LTW*				
	Seq SS	*DF*	*MS*	*F-Value*	*P-Value*	*Seq SS*	*DF*	*MS*	*F-Value*	*P-Value*
U	53.1856	1	53.1856	126.6366	0.0000 *	144.555	1	144.555	15.6525	0.0001 *
U^2	0.0005	1	0.0005	0.0013	0.9716	32.834	1	32.834	3.5552	0.0611
t_{on}	25.4557	1	25.4557	60.6109	0.0000 *	3015.403	1	3015.403	326.5096	0.0000 *
t_{on}^2	3.7557	1	3.7557	8.9424	0.0038 *	703.965	1	703.965	76.2257	0.0000 *
I	230.0952	1	230.0952	547.8642	0.0000 *	23.866	1	23.866	2.5842	0.1098
I^2	30.5381	1	30.5381	72.7122	0.0000 *	1.050	1	1.050	0.1137	0.7364
p	6.6775	1	6.6775	15.8993	0.0002 *	42.032	1	42.032	4.5512	0.0343 *
p^2	12.1405	1	12.1405	28.9069	0.0000 *	89.074	1	89.074	9.6449	0.0022 *
n	80.5072	1	80.5072	191.6902	0.0000 *	82.205	1	82.205	8.9012	0.0033 *
n^2	0.0498	1	0.0498	0.1185	0.7316	70.974	1	70.974	7.6851	0.0062 *
$U\ t_{on}$	2.0289	1	2.0289	4.8309	0.0310 *	0.022	1	0.022	0.0023	0.9614
$U\ I$	0.4963	1	0.4963	1.1816	0.2805	0.002	1	0.002	0.0002	0.9875
$U\ p$	0.1852	1	0.1852	0.4409	0.5087	17.174	1	17.174	1.8596	0.1745
$U\ n$	11.6738	1	11.6738	27.7957	0.0000 *	20.543	1	20.543	2.2244	0.1377
$t_{on}\ I$	19.6637	1	19.6637	46.8200	0.0000 *	331.154	1	331.154	35.8575	0.0000 *
$t_{on}\ p$	1.5079	1	1.5079	3.5903	0.0619	31.508	1	31.508	3.4117	0.0665
$t_{on}\ n$	0.0834	1	0.0834	0.1986	0.6571	249.772	1	249.772	27.0454	0.0000 *
$I\ p$	0.9827	1	0.9827	2.3397	0.1303	53.924	1	53.924	5.8389	0.0167 *
$I\ n$	22.4668	1	22.4668	53.4941	0.0000 *	7.680	1	7.680	0.8315	0.3631
$p\ n$	1.9767	1	1.9767	4.7067	0.0332 *	7.939	1	7.939	0.8596	0.3552
Residual Error	31.4989	72	0.4200	-	-	1579.231	72	9.235	-	-
Total	607.1135	92	-	-	-	7860.098	92	-	-	-

* Indicates the significant term.

Based on Table S1, the regression equations for the *DS* parameter have been simplified for the nonlinear influences of *U* and *n* and the interactions between the factors $U \cdot I$, $U \cdot p$, $t_{on} \cdot p$, $t_{on} \cdot n$ and $I \cdot p$. The equation for the *LTW* parameter has been simplified for the nonlinear influences of *U* and *I* and the interactions between the factors $U \cdot t_{on}$, $U \cdot I$, $U \cdot p$, $U \cdot n$, $t_{on} \cdot p$, $I \cdot n$ and $p \cdot n$.

Table S2. ANOVA table for the *AR* and *HC*.

Source	*AR*					*HC*				
	Seq SS	*DF*	*MS*	*F-Value*	*P-Value*	*Seq SS*	*DF*	*MS*	*F-Value*	*P-Value*
U	73.652	1	73.652	140.514	0.0000 *	0.000462	1	0.000462	60.1940	0.0000 *
U^2	335.608	1	335.608	640.276	0.0000 *	0.000015	1	0.000015	1.9681	0.1619
t_{on}	13.044	1	13.044	24.886	0.0000 *	0.001254	1	0.001254	163.3736	0.0000 *
t_{on}^2	1.467	1	1.467	2.798	0.0950	0.000003	1	0.000003	0.4235	0.5158
I	455.162	1	455.162	868.363	0.0000 *	0.000448	1	0.000448	58.3950	0.0000 *
I^2	10.027	1	10.027	19.129	0.0002 *	0.000000	1	0.000000	0.0000	0.9961
p	1.421	1	1.421	2.710	0.1003	0.000091	1	0.000091	11.8629	0.0007 *
p^2	0.867	1	0.868	1.654	0.1989	0.000029	1	0.000029	3.7909	0.0527
n	89.519	1	89.519	170.786	0.0000 *	0.000100	1	0.000100	12.9802	0.0004 *
n^2	651.002	1	651.003	1241.990	0.0000 *	0.000187	1	0.000187	24.3512	0.0000 *
$U\ t_{on}$	82.329	1	82.329	157.068	0.0000 *	0.000138	1	0.000138	17.9399	0.0000 *
$U\ I$	1.988	1	1.988	3.792	0.0520	0.000227	1	0.000227	29.5641	0.0000 *
$U\ p$	9.986	1	9.986	19.052	0.0002 *	0.000056	1	0.000056	7.3328	0.0073 *
$U\ n$	15.232	1	15.232	29.060	0.0000 *	0.000838	1	0.000838	109.1654	0.0000 *
$t_{on}\ I$	113.437	1	113.437	216.417	0.0000 *	0.000632	1	0.000632	82.3156	0.0000 *
$t_{on}\ p$	102.024	1	102.024	194.642	0.0000 *	0.000720	1	0.000720	93.7787	0.0000 *
$t_{on}\ n$	65.092	1	65.092	124.184	0.0000 *	0.000008	1	0.000008	1.0335	0.3104
$I\ p$	9.964	1	9.964	19.009	0.0001 *	0.000095	1	0.000095	12.3601	0.0005 *
$I\ n$	16.728	1	16.728	31.913	0.0000 *	0.001154	1	0.001154	150.4306	0.0000 *
$p\ n$	1.969	1	1.969	3.757	0.0531	0.000273	1	0.000273	35.5865	0.0000 *
Residual Error	269.943	72	0.524	-	-	0.001841	72	0.000008	-	-
Total	3557.171	92	-	-	-	0.007072	92	-	-	-

* Indicates the significant term.

Based on Table S2, the regression equations for the *AR* parameter have been simplified for the nonlinear influences of t_{on} and *p* and the interactions between the factors $U \cdot I$ and $p \cdot n$. The equation for the *HC* parameter has been simplified for the nonlinear influences of *U*, t_{on}, *I* and *p* and the interactions between the factor $t_{on} \cdot n$.

Table S3. ANOVA table for the *SG*.

Source	*SG*				
	Seq SS	***DF***	***MS***	***F-Value***	***P-Value***
U	1404.7	1	1404.69	10.0654	0.0021 *
U^2	2972.3	1	2972.27	21.2981	0.0000 *
t_{on}	4646.6	1	4646.5	33.2956	0.0000 *
t_{on}^2	2004.0	1	2003.98	14.3597	0.0003 *
I	27153.0	1	27153.05	194.5678	0.0000 *
I^2	7075.3	1	7075.31	50.6988	0.0000 *
p	2086.9	1	2086.89	14.9538	0.0002 *
p^2	315.1	1	315.09	2.2578	0.1367
n	0.0	1	0.02	0.0001	0.9915
n^2	10427.8	1	10427.82	74.7215	0.0000 *
$U\ t_{on}$	229.1	1	229.05	1.6413	0.2037
$U\ I$	533.7	1	533.74	3.8246	0.0538
$U\ p$	1140.2	1	1140.22	8.1703	0.0054 *
$U\ n$	17354.5	1	17354.52	124.3555	0.0000 *
$t_{on}\ I$	16571.5	1	16571.48	118.7446	0.0000 *
$t_{on}\ p$	58.5	1	58.50	0.4192	0.5191
$t_{on}\ n$	0.0	1	0.00	0.0000	0.9989
$I\ p$	136.7	1	136.65	0.9792	0.3252
$I\ n$	2254.6	1	2254.64	16.1559	0.0001 *
$p\ n$	1556.6	1	1556.58	11.1538	0.0013 *
Residual Error	11722.7	72	139.56	-	-
Total	157870.1	92	-	-	-

* Indicates the significant term.

Based on Table S3, the regression equations for the *SG* parameter have been simplified for the nonlinear influences of p and the interactions between the factors $U{\cdot}t_{on}$, $U{\cdot}I$, $t_{on}{\cdot}p$, $t_{on}{\cdot}n$ and $I{\cdot}p$.
